# Oral intake of xanthohumol attenuates lipoteichoic acid-induced inflammatory response in human PBMCs

**DOI:** 10.1007/s00394-022-02964-2

**Published:** 2022-07-20

**Authors:** Finn Jung, Raphaela Staltner, Ammar Tahir, Anja Baumann, Katharina Burger, Emina Halilbasic, Claus Hellerbrand, Ina Bergheim

**Affiliations:** 1 Department of Nutritional Sciences, Molecular Nutritional Science, University of Vienna, Josef-Holaubek-Platz 2 (UZA II), 1090 Vienna, Austria; 2 Department of Pharmaceutical Sciences, Division of Pharmacognosy, University of Vienna, Josef-Holaubek-Platz 2, 1090 Vienna, Austria; 3 Department of Medicine III, Medical University of Vienna, Währinger Gürtel 18-20, 1090 Vienna, Austria; 4 Institute of Biochemistry, Friedrich-Alexander University Erlangen-Nürnberg, Erlangen, Germany

**Keywords:** Xanthohumol, LTA, Hop, TLR2, Inflammation

## Abstract

**Purpose:**

The aim of the study was to determine if xanthohumol, a prenylated chalcone found in Hop (*Humulus lupulus*), has anti-inflammatory effects in healthy humans if applied in low doses achievable through dietary intake.

**Methods:**

In a placebo-controlled single-blinded cross-over design study, 14 healthy young men and women either consumed a beverage containing 0.125 mg xanthohumol or a placebo. Peripheral blood mononuclear cells (PBMCs) were isolated before and 1 h after the intake of the beverages. Subsequently, PBMCs were stimulated with or without lipoteichoic acid (LTA) for 24 and 48 h. Concentrations of interleukin-1β (IL-1β), interleukin-6 (IL-6) and soluble cluster of differentiation (sCD14) protein were determined in cell culture supernatant. Furthermore, hTLR2 transfected HEK293 cells were stimulated with LTA in the presence or absence of xanthohumol and sCD14.

**Results:**

The stimulation of PBMCs with LTA for 24 and 48 h resulted in a significant induction of IL-1β, IL-6, and sCD14 protein release in PBMCs of both, fasted subjects and subjects after the ingestion of the placebo. In contrast, after ingesting xanthohumol, LTA-dependent induction of IL-1β, IL-6, and sCD14 protein release from PBMCs was not significantly higher than in unstimulated cells after 48 h. In hTLR2 transfected HEK293 cells xanthohumol significantly suppressed the LTA-dependent activation of cells, an effect attenuated when cells were co-incubated with sCD14.

**Conclusion:**

The results of our study suggest that an ingestion of low doses of xanthohumol can suppress the LTA-dependent stimulation of PBMCs through mechanisms involving the interaction of CD14 with TLR2. Study registered at ClinicalTrials.gov (NCT04847193, 22.03.2022).

**Supplementary Information:**

The online version contains supplementary material available at 10.1007/s00394-022-02964-2.

## Introduction

Since the discovery of penicillin and its introduction into clinical use revolutionizing the treatment of infections resulting from Gram-positive bacteria, many other antibacterial agents have been introduced in the treatment of Gram-positive bacterial infection. And while many have been successfully used in the beginning, there is a constant struggle with the development of resistance to almost all pharmaceutical due to the adaptability of bacteria (for overview also see [1]). Gram-positive bacteria possess a unique set of protective surface structures that are highly variable and include structures like lipoproteins, peptidoglycans, poly-N-acetyl glucosamine, and wall teichoic acid and lipoteichoic acid (LTA) shown to play important roles in infections (for overview see [2]). For instance, LTA, a membrane-anchored teichoic acid especially found to be indispensable in *Staphylococcus aureus* [3], has been shown to exert host immune recognition and stimulation upon infection through toll-like receptor 2 (TLR2), a receptor either pairing with TLR1 or TLR6 [4]. The LTA-dependent activation of the TLR2-signaling cascade has been shown to go along with a strong induction of the release of cytokines like interleukin-1β (IL-1β), interleukin-6 (IL-6), and tumor necrosis factor α (TNFα) in macrophages and monocytes [5, 6]. It has also been shown that in the circulation LTA binds to lipopolysaccharide binding protein and, similar to lipopolysaccharide, is transferred to CD14 resulting subsequently in the activation of TLR2 [7]. Interestingly, studies suggest that contrasting the findings for the lipopolysaccharide (LPS)-TLR4-signaling cascade, myeloid differentiation factor 2 (MD-2) is not required for the activation of LTA-dependent activation of the TLR2-signaling cascade [8].

Hops (*Humulus lupulus*) has been used for brewing but also in traditional medicine for the treatment of inflammation, pneumonia, and insomnia but also many other diseases for centuries [9]. Throughout the last decades, hop received increasing attention for its unique secondary plant compounds including bitter acids like α-acids and β-acids as well as xanthohumol (for overview also see [10]). Despite having been shown to be poorly absorbed in humans [11] with concentrations peaking 1 h after ingestion followed by a rapid decline [12] xanthohumol has been proposed to possess several beneficial effects with regards to metabolic diseases. Indeed, results of animal studies suggest that xanthohumol at least in parts attenuates the development of non-alcoholic fatty liver disease and insulin resistance but may also be beneficial in the treatment of cancer (for overview see [10, 13, 14]). Recent results of studies by Munoz-Garcia et al. in humans ingesting alcohol-free beer suggest that compounds in beer may dampen immune response of immune cell like macrophages upon LPS stimulation [15]. Results of studies also suggest that xanthohumol interferes with TLR signaling cascades (for overview also see [16]). For instance, in vitro cell and molecular docking studies suggest that xanthohumol can suppress endotoxin-induced TLR4 activation [17, 18]. Chen et al. further suggested that xanthohumol may interfere with endotoxin binding to MD-2 being the co-receptor of CD14 [17]. Furthermore, xanthohumol has been shown to inhibit the expression of inducible nitric oxide synthase (iNOS), nitric oxide (NO), and interferon regulatory factor (IRF) production in in vitro studies in macrophages while showing antifibrotic effects both in vivo and in vitro mediated by hepatic stellate cells [18–20]. However, in most of the studies assessing the effects of xanthohumol doses used were within the pharmacological range i.e. 12 mg/d per person or higher. Such doses cannot be reached through the intake of foods or beverages [21, 22]. If xanthohumol when ingested at low doses corresponding to those found for instance in ~ 500 ml beer (concentrations vary between 0.3 and 3.0 mg/l, depending on the type of beer [23]) after wort boiling, also exerts beneficial effects in humans, e.g., attenuates inflammatory responses, has not yet been assessed.

Starting from this background the aim of the present study was to assess if an acute one-time oral ingestion of 0.125 mg xanthohumol in healthy humans can dampen but not fully diminish the LTA-dependent immune response since dampening but not a full suppression of immune responses in settings of viral or bacterial infections has been suggested and discussed to be superior to a full suppression of the immune response [24].

## Methods

### Study participants

A total of 15 normal weight healthy subjects were enrolled in the study. Written and verbal informed consent was obtained from every participant before the study. The study was approved by Ethics Committee of the University of Vienna, Vienna, Austria (00367) and was carried out in accordance with the ethical standards laid down in the Declaration of Helsinki of 1975 as revised in 1983. Sample size estimation was based on results of previous studies of others [25] assessing the reduction of pro-inflammatory cytokine release in human peripheral mononuclear blood cells using a power analysis (a priori) (Gpower, Version 3.1.9.2). Based on these calculations with an effect size of 1.203, a power of 0.9 and an α-level of 0.05, the sample size of 13 individuals per group was determined. Assuming a drop-out rate of 15–20% we recruited 15 individuals for the study. The present study is part of a bigger study assessing the effect of hop compounds on bacterial toxin-induced inflammation in human. Here, only data assessing the effect of the acute intake of xanthohumol in human health are presented. Study participants did not report food intolerances or food allergies that would require a particular dietary intervention. All study participants confirmed the absence of metabolic diseases, chronic inflammatory diseases or viral and bacterial infections within the last 3 weeks before the study. Furthermore, the use of anti-inflammatory medication was defined as exclusion criteria for the study.

### Intervention study

**Fig. 1:**
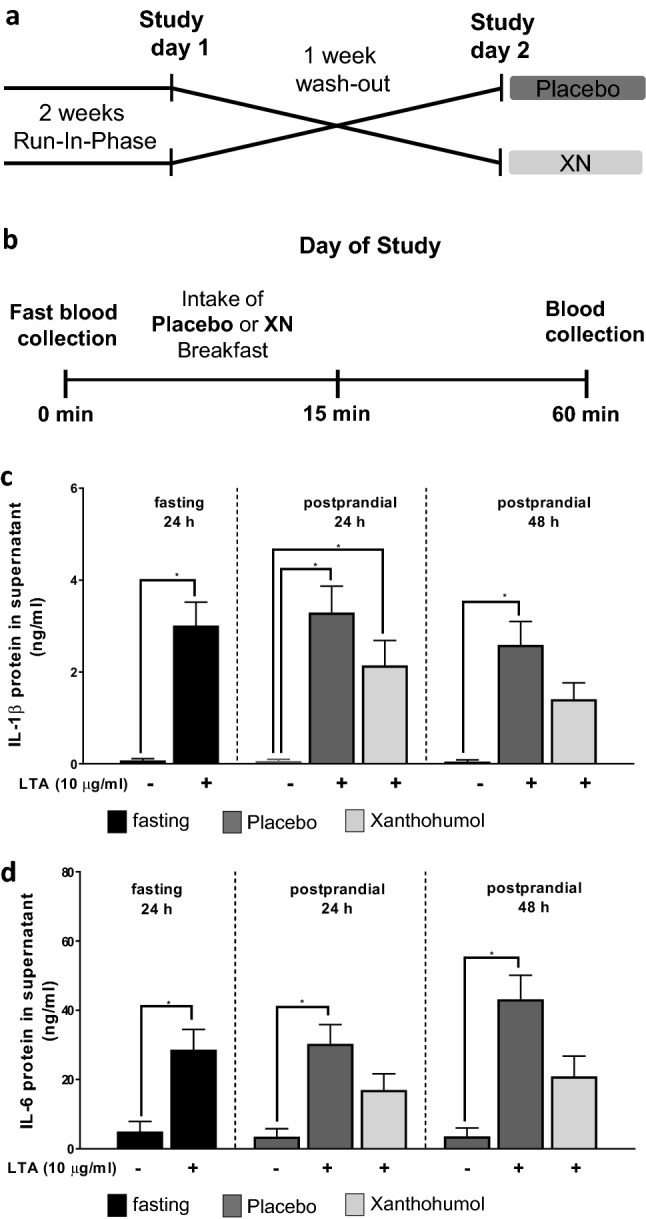
Study design and cytokine concentrations in supernatant of stimulated PBMCs. Graphical illustration of the general study design **a** and on the study days **b** as well as protein concentrations of IL-1β **c** and IL-6 **d** in cell culture supernatant of cells stimulated with 0 or 10 µg/ml LTA isolated from healthy study participants receiving either a placebo or xanthohumol. *IL* interleukin, *LTA* lipoteichoic acid, *PBMC* peripheral blood mononuclear cell, *XN* xanthohumol. Data are expressed as means ± SEM. *p < 0.05. Figure was created with Microsoft PowerPoint (**a**, **b**) and GraphPad Prism 7 (**c**, **d**)

The study design is summarized in Fig. 1a and b. In brief, after a 2-week-long wash-out phase, during which participants were asked to refrain from the consumption of hops containing food and beverages, a fasting blood sample was obtained from all participants. Study participants were then randomly and single-blinded assigned to either consume 0.125 mg xanthohumol (XanthoFlav, generous gift from Hopsteiner GmbH, Germany) or a placebo. The xanthohumol (XanthoFlavTM) used in this study was a generous gift from Hopsteiner GmbH. XanthoFlav^™^ is a natural product from hops and consists mainly of xanthohumol (75%) and other prenylated flavonoids (25%) occurring naturally in hops (for further details also see: https://www.hopsteiner.com/wp-content/uploads/2021/12/26_21_ls_xanthoflav.pdf). The beverage containing xanthohumol was prepared as follows: 0.125 mg xanthohumol (XantoFlav, Hopsteiner GmbH, Germany) was added to 10 ml water followed by the addition of a thickener (Thicken Up Clear, Nestlé GmbH, Swiss), 70 mg skim milk powder and lemon flavor (Pepsico Inc., United States). The placebo was prepared by adding thickener (Thicken Up Clear, Nestlé GmbH, Swiss), 70 mg skim milk powder and lemon flavor (Pepsico Inc., United States) to 10 ml water. The xanthohumol drink or the placebo was consumed once before a light breakfast consisting of two medium sized pretzels and 30 g butter. Participants were asked to consume the breakfast and study beverages within 15 min. Sixty minutes later, blood was collected and peripheral blood mononuclear cells (PBMCs) were isolated as detailed below. The timing of blood sampling was chosen based on studies by Legette et al. and Ferk et al. [12, 26]. In this dose–response study the highest concentration of xanthohumol was detected 1 h after the oral intake in healthy individuals, whereas no xanthohumol was detected 4 h after the intake. Following a second wash-out phase of 7 days during which the participants were again asked to refrain from hop contained foods and beverages, the intervention was repeated in a cross-over design with each participant (see Fig. 1a for graphical illustration of study design).

### Isolation and culture of PBMCs

PBMCs were isolated from whole blood samples using the Vacutainer^®^ CPT^™^ System following the manufacturer instructions (Becton Dickinson GmbH, Germany). 500.000 cells were suspended in ‘complete’ medium (RPMI-1640 medium (Sigma–Aldrich Corp., US, 10% fetal bovine serum (Pan-Biotech GmbH, Germany)), 100 μg/ml streptomycin and 100 U/mL penicillin (Pan-Biotech GmbH, Germany)). After 1 h in ‘complete’ medium cells were challenged either with 0 or 10 µg/ml LTA for 24 h and 48 h, respectively. Cell culture supernatant and cells were harvested and stored at − 80 °C until further use.

### Isolation of monocytes and lymphocytes

To isolate monocytes and lymphocytes, PBMCs were isolated as detailed above. PBMCs were then transferred on an iso-osmotic Percoll solution consisting of Percoll (density: 1.131 mg/ml (Sigma–Aldrich Corp., US), ‘complete’ medium and 10 × PBS to obtain lymphocytes and monocytes as detailed by Menck et al. [27]. Lymphocytes and monocytes were then spotted on a glass slide via cytospin and stained with DAPI (Sigma–Aldrich Corp., US).

DAPI staining was detected using DAPI filter system included in the microscope (Leica DM6B). Binding of xanthohumol to cells was determined as detailed before by others [28, 29]. In brief, spotted cells were excited at 480/40 nm and emission was detected at 530 nm to detect autofluorescence of xanthohumol. Lymphocytes and monocytes of participants before the ingestion of xanthohumol were used as controls. In advance, autofluorescence of xanthohumol was validated in a pilot study using PBMCs obtained from 4 healthy participants. In this study cells were incubated with 0–8 μg/mL xanthohumol for 0–4 h and in 5–10% of cells autofluorescence was detectable (data not shown).

### Cell culture experiments with hTLR2 transfected HEK cells response

A commercially available reporter gene assay with TLR2 transfected HEK293 cells was used to assess effects of xanthohumol on CD14/TLR2 signaling (InvivoGen, France, Cat.Number: hTLR2 = hkb-htlr2,). These cells are transfected with human TLR2 and also human CD14. Cells were grown according to the instructions of the manufacturer. In brief, cells were grown in a humidified, 5% carbon dioxide atmosphere using DMEM media (Pan-Biotech, PAN-Biotech GmbH, Aidenbach, Germany) containing 10% fetal bovine serum (PAN-Biotech GmbH, Aidenbach, Germany) and 1% penicillin/streptomycin (PAN-Biotech GmbH, Aidenbach, Germany) up to 80% confluence. Cells were then challenged with 0 or 10 µg/ml LTA in the presence of 0–8 µg/ml xanthohumol dissolved in HEK BlueTM Detection medium (InvivoGen, France) for 12 h. Color changes of medium being indicative of ligand concentration and binding were determined at 655 nm. In a second set of experiments, cells were again grown to 80% confluence and challenged with 0 or 10 µg/ml LTA in the presence of 0 or 3 µg/ml xanthohumol and 0–1000 ng/ml sCD14. The xanthohumol dose used in this experiment was determined in the first set of experiments. After 12 h, color changes of medium were determined at 655 nm.

### Western blot

Cell culture supernatant and cell lysate was separated in a polyacrylamide gel and was transferred on a polyvinylidene difluoride membrane (Bio-Rad Laboratories, Hercules, CA, USA). After blocking, membranes were incubated for 1 h with a specific monoclonal CD14 antibody (#56082S, Cell Signaling Technology Inc., US) or TLR2 antibody (#17,236–1-AP; Proteintech Group Inc., United States) followed by a 1 h incubation with the appropriate secondary antibody (#7074S, Cell Signaling Technology Inc., US). Bands were detected using Super Signal West Dura kit (Thermo Fisher Scientific, Waltham, MA, USA). Densitometric analysis of bands was performed using Image Lab 6.0 Software (Bio-Rad Laboratories, Hercules, CA, US).

### Enzyme-linked immunosorbent assays (ELISA’s)

IL-1β and IL-6 protein concentrations were analyzed in cell culture supernatant using commercially available ELISA kits (IL-1β: Sigma–Aldrich Corp., US; IL-6: Bio-Techne Corp., US).

### Relative quantification of xanthohumol using UHPLC-HRMS

For protein precipitation, plasma samples were incubated with pre-cooled acetonitrile (ACN) and stored at − 20 °C for 2 h. Samples were centrifuged and supernatant was concentrated by vacuum centrifugation. Samples were subsequently reconstituted in Acetonitrile for further analysis. Xanthohumol in plasma of participants was then measured by ultra-high-performance liquid chromatography and high resolution mass Spectrometry (UHPLC-HRMS).

UHPLC: EXIONLC AD SYSTEM (AB Sciex, Darmstadt, Germany) with a reversed-phase C18 column (ACQUITY UPLC CSH C18 Column, 130 Å, 1.7 µm, 2.1 mm X 100 mm, Massachusetts, USA) was used for measurement. Mobile phase A (H_2_O/FA, 99.98:0.02) and mobile phase B (ACN/ /FA, 99.98:0.02) were degassed prior to usage. A 7 min binary gradient with flow rate set to 350 μL/min was applied as follows: 0–1 min, 50% mobile phase B; 1–4 min, 50 − 99% mobile phase B; 4–5 min, 99% mobile phase B; 5.1–7 min re-equilibration with 50% mobile phase B). From each sample 1 μl were injected followed by a blank injection to ensure proper column washing and equilibration.

The mass spectrometric detection (HRMS) was performed using a turbo ion source ESI X500 QTOF mass spectrometer (AB Sciex, Darmstadt, Germany), 500 °C heater temperature, with the ion source gas1 set to 30 psi and ion source gas2 set to 30 psi. Curtain gas was set to 45 psi and spray voltages of ± 5.0 kV were applied to achieve negative ion mode ionization. TOFMS scans were performed with an m/z range from 100 to 1500. TOF MS/MS scans of the three most abundant ion were achieved through collisional dissociation fragmentation at 35 V collision energy, 0.1 accumulation time and −80 V decluttering potential. MRM-hr method was used to specifically quantify the Xanthohumol peak. The precursor ion of xanthohumol was identified as [M–H] = 353.1389 Da ± 3 ppm, and two product ion were separately monitored, namely: 239.0405 Da ± 10 ppm as a qualifier and 119.0502 Da ± 10 ppm as a quantifier. For the fold changes calculation, each sample was technically replicated and the normalized area under the curve was used to perform the relative quantification. An authentic reference of xanthohumol was used to develop the parameters for the MRM-hr method (retention time, exact masses for precursor and product ions and Collision energies).

### Statistical analyses

Data are presented as means ± standard error of the means (SEMs). Grubb’s test was performed before statistical analysis to identify outliers (GraphPad Prism Software, USA). Friedman-Test with Dunn’s multiple comparison test was used to determine statistically significant differences between interventions (GraphPad Prism Software, USA) and *p* ≤ 0.05 was selected as the level of significance.

## Results

### Effect of the consumption of low-dosed xanthohumol on LTA-induced inflammatory response of isolated PBMCs

Anthropometric and health characteristics of study participants as well as routine laboratory parameters are summarized in Table 1. In total 14 healthy, normal weight subjects (5 women und 9 men) aged 21–30 years were analyzed as 1 male participant had to be excluded due to metabolic abnormalities detected during screening. Somewhat in line with the findings of others [12, 26] 1 h after the ingestion, xanthohumol and to a lesser extend xanthohumol metabolites were detected in plasma after the ingestion of the study drink containing xanthohumol (see Supplemental Fig. 1).

**Table 1:** Anthropometric and health characteristics of study participants

Parameter	Healthy participants
Sex (m/f)	9/5
Age (years)	25.6 ± 0.8
Body weight (kg)	66.1 ± 2.8
Height (m)	1.72 ± 0.03
BMI (kg/m^2^)	22.2 ± 0.5
Blood pressure	
Systolic (mmHg)	124.2 ± 3.9
Diastolic (mmHg)	76.2 ± 2.4
Fasting glucose (mg/dl)	88.5 ± 2.4
Uric acid (mg/dl)	5.0 ± 0.3
AST (U/L)	29.8 ± 5.8
ALT (U/L)	34.9 ± 11.2
γ-GT (U/L)	14.7 ± 1.4
Cholesterol (mg/dl)	169.3 ± 10.0
HDL cholesterol (mg/dl)	60.8 ± 4.0
LDL cholesterol (mg/dl)	93.2 ± 8.2
Triglycerides (mg/dl)	77.1 ± 8.1
CRP (mg/dl)	0.07 ± 0.02

*AST* aspartate aminotransferase, *ALT* alanine aminotransferase, *CRP* c-reactive protein, *γ-GT* γ-glutamyltransferase, *HDL* high density lipoprotein, *LDL* low density lipoprotein

Data are expressed as means ± SEM

As expected, after 24 h, LTA stimulation of PBMCs isolated from fasting blood resulted in a significant ~ 40-fold increase in IL-1β and a significant ~ sixfold increase in IL-6 protein levels in cell culture media compared to unstimulated cells. In line with these findings, LTA-stimulation of PBMCs for 24 h and 48 h, respectively, isolated after the ingestion of placebo, resulted in a significant ~ 54-fold (24 h) and a ~ 50-fold (48 h) increase of IL-1β and a significant ninefold (24 h) and a ~ 12-fold (48 h) increase of IL-6 protein levels in cell culture media compared to unstimulated cells. IL-1β protein levels in cell culture supernatant of PBMCs isolated from subjects after ingesting xanthohumol stimulated for 24 h with LTA were significantly higher by ~ 35-fold. In contrast, when these cells were stimulated for 48 h, IL-1β protein levels neither differed from those determined in media obtained from unstimulated cells or stimulated cells. Furthermore, after 24 and 48 h of stimulation with LTA, respectively, concentration of IL-6 protein obtained from PBMCs of subjects after ingesting xanthohumol neither differed from that determined in medium of unstimulated cells nor from that obtained from cells isolated after subjects had ingested placebo and stimulated with LTA (Fig. 1c, d).

As data were similar after 24 and 48 h, respectively, in Fig. 1c and d, only protein levels of IL-1β and IL-6 measured in media collected from PBMCs obtained from fasted participants that were stimulated with 10 ng/ml LTA for 24 h are shown. Also, as protein levels of the two cytokines in media obtained from vehicle stimulated cells were similar before the light breakfast regardless of drink ingested after breakfast, only data obtained from PBMCs collected after the ingestion of the placebo are shown in the graphs.

### Binding of xanthohumol to PBMCs

**Fig. 2:**
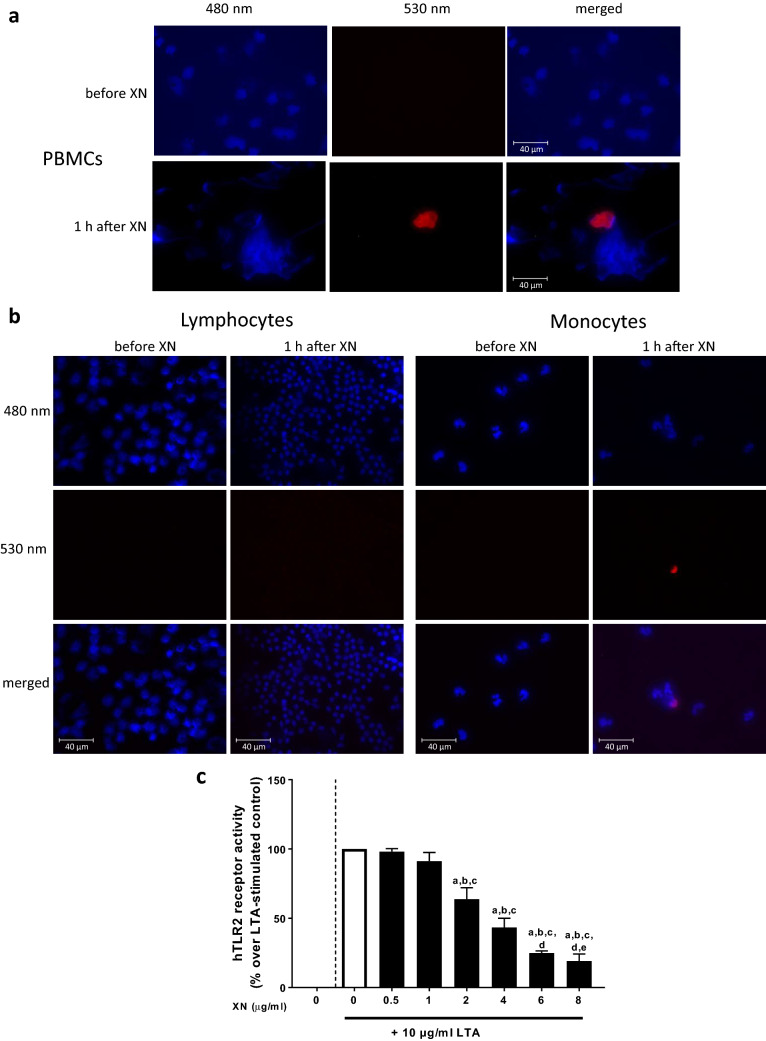
Fluorescence imaging of PBMCs isolated from study participants as well as hTLR2 receptor activities of HEK293 cells stimulated with 0–8 μg/ml xanthohumol for 12 h. Fluorescence imaging of PBMCs (**a**) as well as lymphocytes and monocytes (**b**) obtained from study participants before and after oral ingestion of xanthohumol (magnification: 630×) and hTLR2 receptor activities from HEK293 cells co-incubated with lipoteichoic acid (10 μg/ml) and increasing concentrations of xanthohumol (0–8 μg/ml) (**c**). *hTLR2* human toll-like receptor 2, *LTA* lipoteichoic acid, *PBMC* peripheral blood mononuclear cell, *XN* xanthohumol. Data are expressed as means ± SEM. *p < 0.05. Figure was created with Leica LAS X (**a**, **b**) and GraphPad Prism 7 (**c**)

To determine if the immune suppressive effects of xanthohumol on LTA stimulation of PBMCs were related to a direct binding of xanthohumol to cells, PBMCs were spotted on microscope slides and exposed to 480 nm as it has been shown before by others that xanthohumol is autofluorescent at this wave length [28, 29]. Upon exposure to 480 nm, no fluorescence light emission was detected in cells obtained after the ingestion of placebo while ~ 10% of PBMCs of subjects after the ingestion of the xanthohumol-enriched drink were found to emit light suggesting that xanthohumol was bound to or taken up by specific blood cells (Fig. 2a). Indeed, in a subsequent fractioning of blood cell populations we found that fluorescence was only prevalent in the monocyte fraction, while no fluorescence was detected in the lymphocyte fraction (Fig. 2b).

### Effect of xanthohumol of LTA-dependent activation of TLR2 transfected HEK blue cells

To determine if the effects of xanthohumol found in the cell stimulation experiments might have resulted from blocking the activation of TLR2, HEK blue cells transfected with TLR2 and CD14 were challenged with LTA (10 μg/ml) in the presence of increasing concentrations of xanthohumol (see Fig. 2c). Xanthohumol attenuated the activation of cells in an almost dose-dependent manner, with 8 µg/ml of xanthohumol almost completely attenuating the effects of LTA.

### Effect of xanthohumol intake on TLR2 and CD14 protein levels in isolated PBMCs

**Fig. 3:**
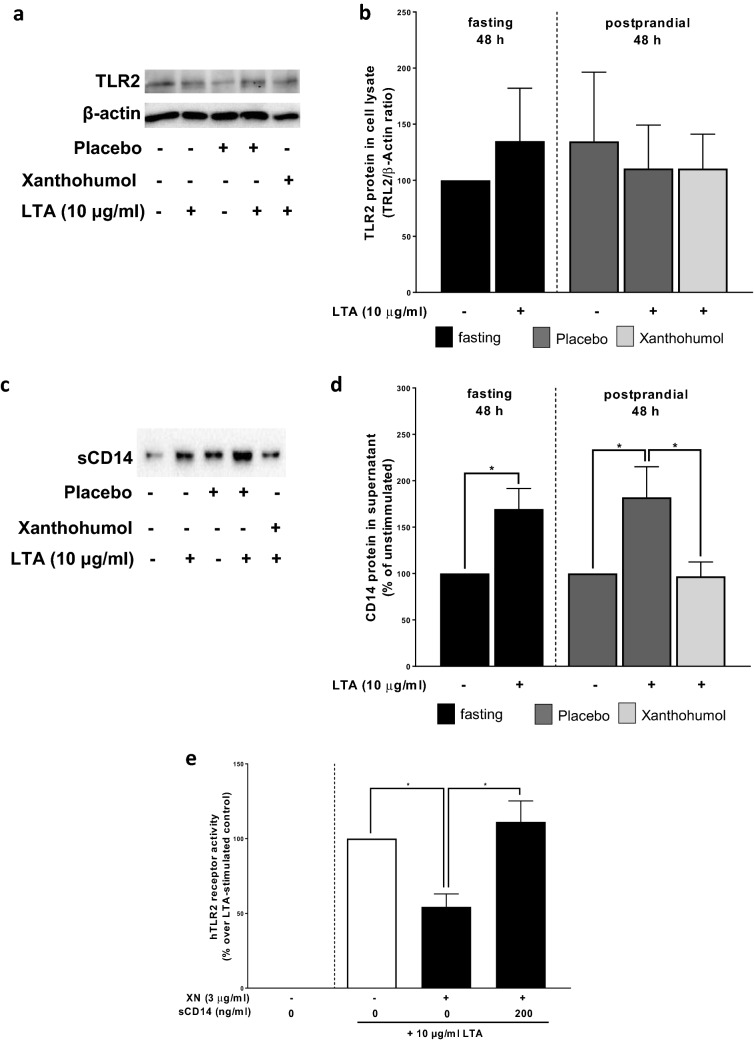
Protein concentration of TLR2 in cell lysate and sCD14 in cell culture supernatant of LTA-stimulated PBMCs as well as effect of sCD14 on LTA-stimulated HEK293 cells co-incubated with xanthohumol. Representative blots (**a**) and densitometric analysis of TLR2 western blot (**b**) in cell lysate as well as representative blots (**c**) and densitometric analysis of sCD14 western blot (**d**) in cell culture supernatant of LTA-stimulated (10 μg/ml) PBMCs obtained from study participants either receiving a placebo or xanthohumol. hTLR2 receptor activities of HEK293 cells incubated with xanthohumol (3 μg/ml) and sCD14 (200 ng/ml) (**e**). *LTA* lipoteichoic acid, *hTLR2* human toll-like receptor 2, *sCD14* soluble cluster of differentiation 14, *PBMC* peripheral blood mononuclear cell, *XN* xanthohumol. Data are expressed as means ± SEM. *p < 0.05. Figure was created with Bio-Rad Image Lab 4.0 (**a**, **c**) and GraphPad Prism 7 (**b**, **d**, **e**)

To further determine, if the effects found after an oral ingestion of xanthohumol were related to changes in the CD14/ TLR2-signaling cascade in vivo, too, we next determined TLR2 protein levels in cell lysates as well as sCD14 in cell supernatant of cells isolated from study participants and stimulated with or without LTA for 48 h. The stimulation of PBMCs with LTA for 48 h had no effects on TLR2 protein levels in PBMCs isolated from fasting blood or cells isolated postprandial regardless of drinks ingested (see Fig. 3a and b). In contrast, protein levels of sCD14 in cell supernatant were significantly higher in cells isolated from fasting blood. A similar increase in levels of sCD14 was also found in supernatant of cells stimulated with LTA isolated from subjects after the consumption of the placebo. In contrast, in supernatant obtained from LTA-stimulated cells isolated after the ingestion of the xanthohumol-enriched drink, the sCD14 levels were at the level of controls (xanthohumol vs. placebo *p* < 0.05) (Fig. 3c and d).

### Effect of sCD14 on xanthohumol-dependent attenuation of LTA-induced inflammatory response of PBMCs

To further determine if (s)CD14 is critical in mediating the xanthohumol-dependent suppression on the LTA-induced stimulation of PBMCs, TLR2 transfected HEK blue cells were challenged with increasing sCD14 concentrations in the presences of 0 or 3 µg/ml xanthohumol. The addition of xanthohumol resulted in a ~ 50% suppression of the LTA-dependent activation of cells. The addition of 200 ng/ml sCD14 to the cell media resulted in a `recovery´ of the LTA-dependent activation of cells in an almost dose-dependent manner (see Fig. 3e) which was even enhanced compared to the stimulation of LTA alone when higher concentrations of sCD14 were employed (data not shown).

## Discussion

### The oral intake of xanthohumol attenuates the LTA-dependent stimulation of PBMCs

Bacterial infections are common and can lead to life-threatening illness requiring sometimes long lasting and cost intensive therapies. Antibiotics are still the first option of choice in the treatment of most bacterial disease; however, over time, antibiotic resistance often limits therapeutic options [30]. In the present study, we assessed the effect of the acute intake of low doses of xanthohumol on LTA-dependent immune responses in isolated PBMCs of healthy young humans. Doses of xanthohumol used in the present study were based on those found in ~ 500 ml beer after wort boiling, where most of the xanthohumol is isomerized to isoxanthohumol [31]. Despite using markedly lower doses than other studies showing anti-inflammatory effects in rodents [20, 32, 33], in the present study, xanthohumol was detected in plasma after the ingestion of the xanthohumol containing drink and LTA-dependent activation of PBMCs was markedly lower after the ingesting of the xanthohumol containing beverage than the placebo. However, due to the variability between subjects cytokine levels between the placebo and xanthohumol group did not differ significantly when compared to each other. Still, immune responses were not completely reduced to that of unstimulated cells. Indeed, it has been suggested by others before that a total suppression of immune responses to viral or bacterial challenges may be counterproductive and may even enhance the severity of the disease and recovery [24]. Furthermore, results of the present study suggest that within 1 h of its ingestion xanthohumol is taken up into the blood stream where it at least in part binds to monocytes while not being detected in T- and B-cells. Taken together, these data suggest that the intake of low doses of xanthohumol may dampen the immune response to LTA in humans and that these effects may be related to a binding or an uptake of xanthohumol by monocytes. However, as in the present study, PBMCs were only exposed to LTA ex vivo, further studies are needed to determine if effects are also present in humans suffering from an infection with gram positive bacteria.

### How does xanthohumol attenuate the LTA-dependent stimulation of PBMCs?

It has been shown before that xanthohumol can bind to cell membranes [34] and may be taken up by different cell types such as hepatocytes, stellate cells and Caco-2 cells [11, 28]. However, to our knowledge it has not yet been clarified how this uptake is mediated and whether blood cells also can take up xanthohumol. In the present study, we found a fluorescence signal in the monocyte subfraction of isolated PBMCs 1 h after the ingestion of xanthohumol, suggesting that xanthohumol either bound only to these cells or was only taken up by them. Interestingly, no fluorescence was detected in the T- and B-cell fraction. These data suggest that xanthohumol may selectively bind to certain immune cells or protein(s) located in their cell membrane. Indeed, it has been suggested by the results of others that xanthohumol may suppress endotoxin-dependent activation of TLR4 through MD-2-dependent mechanisms and that herein a binding to MD-2 might be the underlying mechanism [17]. Shimazu et al. already showed more than 20 years ago that MD-2 is required for the LPS-dependent signaling of TLR4 [35]. It has further been shown that LPS is bound to CD14 and transferred to TLR4/ MD-2 and that CD14 is critical for the recognition of LPS by TLR4 and MD-2 [36, 37]. Studies further suggest that MD-2 might also be involved in TLR2-dependent signaling [38] and that it is also physically associated with TLR2. However, the association with TLR2 is thought to be weaker than with TLR4 [39]. Indeed, more recent studies suggest that MD-2 might play an inferior role in the activation of TLR2 signaling [40], while CD14 has been suggested to be an essential factor in Gram-positive bacterial toxin dependent activation of TLR2 [41]. In the present study, using commercially available HEK293 cells transfected with TLR2 and CD14 but not expressing MD-2, we found that xanthohumol, blocked the LTA-dependent activation of TLR2 in an almost dose-dependent manner. We further showed that these effects of xanthohumol can be attenuated by sCD14 in an almost dose-dependent manner. Furthermore, TLR2 protein levels were not altered in LTA-stimulated cells obtained from study participants 1 h after the ingestion of xanthohumol or placebo, while the concentration of sCD14 was only altered in cells obtained after the ingestion of the placebo. It has been suggested by the results of others, that CD14 is `shed´ from the cell surface [42] and that this may be critical in the inflammatory response to bacterial toxins [43]. Taken together, these data suggest that xanthohumol attenuates LTA-dependent activation through CD14-dependend mechanisms. However, our results by no means preclude that xanthohumol may also affect other cellular signaling cascades, especially, when consumed at higher doses and/ or over an extended period of time. Still, our results suggest, that even non-pharmacological doses like the once used in the present study, xanthohumol may at least temporally attenuated the LTA-dependent activation of the CD14/ TLR2-signaling cascade.

### Limitations

Our study is not without limitations that need to be considered when interpreting the data. For instance, in the present study, cell stimulation experiments were carried out ex vivo. Accordingly, further studies are needed to determine, if effects alike are also present in patients suffering from bacterial infections with Gram-positive bacteria. Also, xanthohumol was only ingested once and PBMCs were isolated 1 h after the ingestion. Therefore, from our results no propositions regarding long-term effects of xanthohumol on bacterial toxin-triggered immune response can be made. Furthermore, as we only determined effects 1 h after the ingestion of xanthohumol no predications regarding persistence of the effects found can be made. Also, in the present study, we only assessed effects in healthy, normal weight young men and women. If effects alike are also found in overweight and/or older individuals needs to be determined in future studies.

## Conclusion

**Fig. 4:**
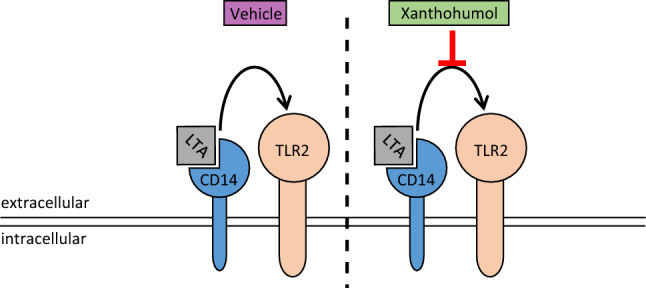
Schematic summary of the study results. *CD14* cluster of differentiation 14, *LTA* lipoteichoic acid, *TLR2* toll-like receptor 2. Figure was created with Microsoft PowerPoint

In summary, results of the present study suggest that an oral ingestion of low doses of xanthohumol may be sufficient to alter LTA-dependent immune responses of monocytes in peripheral blood of healthy humans. Our results further suggest that the beneficial effects of xanthohumol are related to a suppression of the CD14-/TLR2-dependent activation of cells (graphical summary of study results: see Fig. 4). Further studies are needed to determine the duration of this suppressive effect, as well as exact molecular mechanisms involved.

## Supplementary Information

Below is the link to the electronic supplementary material.

Supplementary file1 (PDF 182 KB)

## Abbreviations

ACN: Acetonitrile
AST: Aspartate aminotransferase
ALT: Alanine aminotransferase
CRP: C-reactive protein;
γ-GT: γ-Glutamyltransferase
HDL: High density lipoprotein
IL-1β: Interleukin-1β
IL-6: Interleukin-6
iNOS: Inducible nitric oxide synthase
IRF: Interferon regulatory factor
LDL: Low density lipoprotein
LPS: Lipopolysaccharide
LTA: Lipoteichoic acid
MD-2: Myeloid differentiation factor 2
NO: Nitric oxide
PBMC: Peripheral blood mononuclear cell
sCD14: Soluble cluster of differentiation 14
TLR: Toll-like receptor
XN: Xanthohumol
