# Supplementary material for: Oral intake of xanthohumol attenuates lipoteichoic acid-induced inflammatory response in human PBMCs
Source: Eur J Nutr. 2022 Jul 20;61(8):4155–66. doi: 10.1007/s00394-022-02964-2 (PMC9596557; doi:10.1007/s00394-022-02964-2)
Supplement: Supplementary file 1 — Supplementary file1 (PDF 182 KB) [file 394_2022_2964_MOESM1_ESM.pdf]

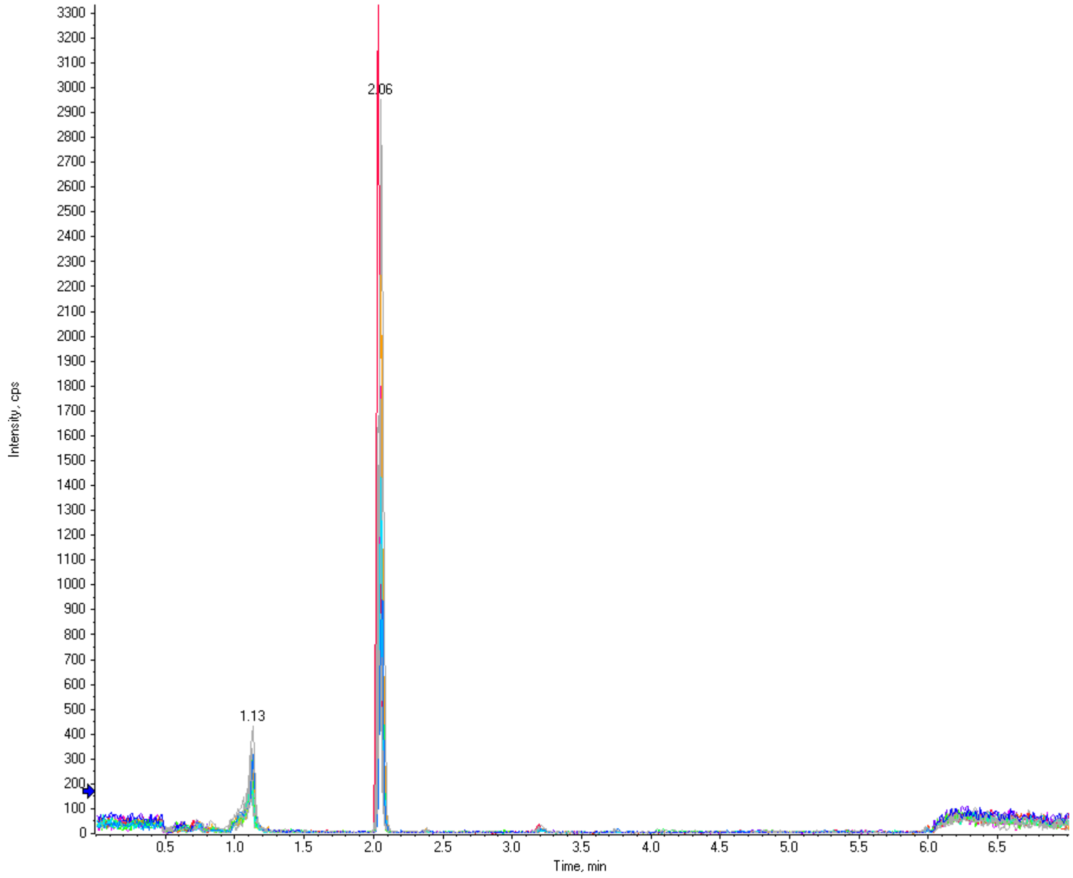

Supplemental Fig. 1: Overlay of UHPLC-HRMS chromatograms representing xanthohumol signals detected as the product ion 119.0504 Da (Rt= 2.04 min) in plasma of subjects (n=14) 1 h after the ingestion of 0.125 mg xanthohumol.
